# Supplementary material for: circRNA432 enhances the coelomocyte phagocytosis via regulating the miR-2008-ELMO1 axis in Vibrio splendidus-challenged Apostichopus japonicus
Source: Commun Biol. 2023 Jan 28;6:115. doi: 10.1038/s42003-023-04516-8 (PMC9884281; doi:10.1038/s42003-023-04516-8)
Supplement: Supplementary file 2 — Supplementary information [file 42003_2023_4516_MOESM2_ESM.pdf]

Supplementary Table 1 miRanda predicts the binding sites of circ432 and miRNAs.

circRNA	miRNA	Score	Energy (kcal/mol)	Range
circ432	miR-2008	144	-21.42	1296-1318
circ432	miR-2008	140	-21.57	238-259
circ432	miR-137	156	-13.37	845-866
circ432	miR-137	152	-13	2145-2166
circ432	miR-137	141	-12.7	1863-1885
circ432	miR-9	150	-14.77	1737-1758

Supplementary Table 2 miRanda predicts the binding sites of miR-2008 and phagocytosis-related genes.

miRNA	Target gene	Score	Energy (kcal/mol)	Range
miR-2008	cell division cycle 42	155	-10.92	2050-2071
miR-2008	beclin-1	147	-13.69	2843-2864
miR-2008	interleukin-1 receptor-associated kinase	150	-14.77	163-185
miR-2008	Engulfment and cell motility protein 1	156	-15.77	1797-1819

circc432: AJAPscaffold432:393652|396009

parent gene: EVM gene 393624 396158

Three exons: EVM exon 393624 393855

EVM exon 394584 394913

EVM exon 395881 396158

CTGTTTCACCCGATGAGATATCAATAGAGGATGAACTGTCTAAGATGGATAACAGGGATGATGGGACTTCTAACATTACACTCAGATCAAGTTTCCGTGTCCTG → Exon 1
AGCCAGTCCATACACCATGTCACTCTGGAATGTAGGGTAGTAGATGAGAATGATGGTTCACAACATGATGTCACTACAGTAGAGGTTCTCTTTGATAAAAGTG
AGGCTCATGAATCTCTTGCTTTTATATTTTGGTATGACATAGTAGGGCTGATTTTGAATGATTTTTCTAGATGTTTACTGGTATTAGCTATCCTTCCATTTTTAATTT
CTAAAGGGCAAACTTACTAGTCTGAAAACTAAGGTATAGGCATGAATGGAGAACAAAAACAAATGTCACATACACATGTTCCTTCAGACAGTGGAACTGCAG
TGTCTATGTTTTTAACTGTCATCAAATTATTATTATGAAAGGGCAGGACACATGAAGAAAAACATTTGCATAATCACTCAGGAACTCTACCAAATAATGTTGAA
CCATTCGCAAATACTGAGGGCAACTTGACACAACTCTGTTCTTGGATGTTGGATGCTCATCTCATATTGTATACATGAAAATGAGTAACCTGTTGTGTTTGGTG
GAGAGAAAGTGATATTTAATGTTATAAATGGTGATGGCTGTAGTTTGAAGTTTCCTTACTGTAAATAAAACCAAGTGATTCTTTGGGAGTATTCAGCTTATAAA
ACTGTGAGGCTACTATTCTAGAAGAGCTAGAGTAGAATAAATAAAGTCATCTTCCTGAACTGCTCTGATGATAGTGCTCTGCCCAACACTTATTAAATTCATGT
CCTAGTTGAAAACAAGTATATTTTAAGGCAATATCACATACAGTAGTAGATTGAAAATCAACAGTTTGAATGATTTTGTTGTTTTGATCTCCTGTTCAGACTTCC
CAGTGGTAAATGGATTGGTCCATCAGCAACATGTGGTCCTGAATGTGAAAAGAGAAGACAAACTGACTTGCACCATGAATAGAGTCTCACCAACTATGGTTT
TAGAATGGGTCTTCACTCCACCTGTTCCACCCAATGAGATATCAATGGACAATGAACTGTCTAAGACGGATAACAGGGACGGGACTTCTAACATTACACTCAG → Exon 2
CTCAAGTTTCCGTGTCCTGAGTCAGTCCATACACTATGTCACTCTGGAATGTAGGGTAGTAGATGAGAATGATGGTTCACAACATGATGTCACTACAGTAGAG
CTTCTCTTTGATAACAGTAAGGCTCATTAATTTTTATGCTTTAATATTTTTGTTTGACTTAGTAGGGCTGATTTTGAAGGAATTTTCCAGATGTTCCTGGTATTACC
TATCCTTCCATTTAAAATTTCTAAGGACAAACTTACTAGTCTGAAAACTAAGGTCTGGTCATGAACGGAGAACAAAACACAAAGTCACATACACATGTTCCTT
CAGACAGTGGATCTGCAGTGTCCATGTTTATAACAGTCATTAAATTATCATTACAAAAGGGTTGTGGATTGTGTACAGTAACAAAGACAGAACACATTAAGGA
AACATTTGCATAATCGCTCAAGAATTCTGTTCAGGAAGATGATCATGTAGTTCTACTCTAGCTCTTCTTGGAAAGTAGCCTCACATTGTTACAAGCTGAATACT
CCCGAAGAATCACTTGGTTTATTTACAGTAAGGACACTGCAAACTACAGCAATCACCAGTTATAAAATTAAATATCACTTTCTCTCCACCAAAGACAATAGGTT
ACTCATGTTGATGTTAAATATGGGGTGAGTAATCCAATTCCAAGAACAGAGTTGTGTCAAGTTGCCCTCAGTATTTGAGAAATGGTTCAACTTTATTTGGTAGA
GTTCCTAAGCGATTATGCAAATGTTTTTCTTCATTTGTGCTGTCTTTGTTACTATACACAATCCACTGCCCTTTTGTAATAATAATTTGATGACATTTAAAAACAT
GGAGACTGCAGTTCCACTATCTGAAGGAACATGTGTATTCAGCTTATATTGTGAGGCTACTATCCAAGAAGAGCTAGAATAGAAGAACTGCTGTATGATCATCT
TCCTGAACAGCTTTGATGATAGTTTCCCGTCCTACATTAATTAATTTTATGACCTAGTTGAAAACAAGTATAGTTTTAGGCAATTTCACATACAGTAGTAGTTTG
AAAATCAAAACATTGAATGATTTTGTTTTGATCCACTGTTCAGACTTCCCTGTGGTGAATGGATTGGTCCATCAGCAACATGTGGTCCTGAAGGTGAACAGAG → Exon 3
AAGACAAACTGACTTGCACCATGAATAGAGTCTCACAAACTATGGTTTTAGAATGGGTCTTCACTCCAC

Supplementary Figure 1 Full length of circ432. Exon sequences are shown in red.

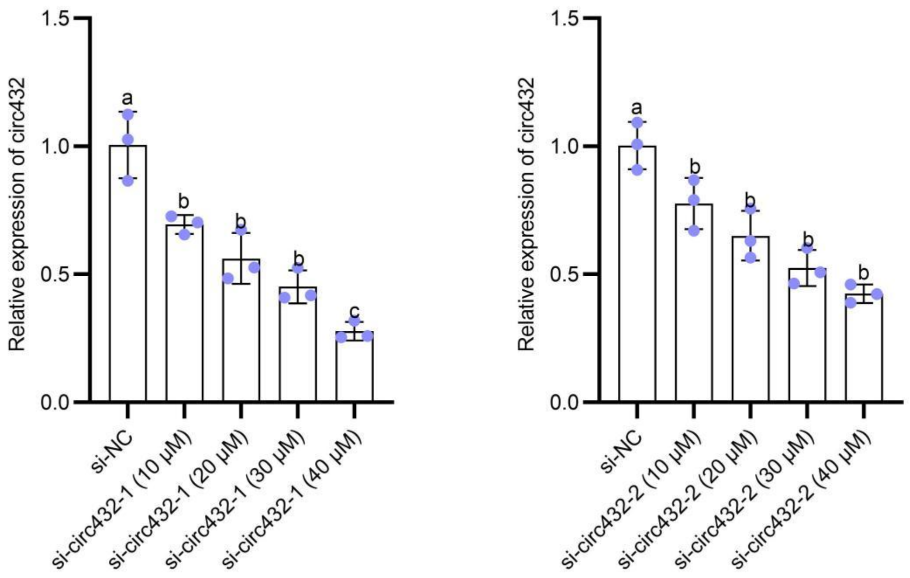

Supplementary Figure 2 The relative abundance level of circ432 after siRNA transfection (si-circ432-1 and si-circ432-2) at different concentrations *in vivo* was detected by qPCR. All data represented the mean ± SD from three independent triplicated experiments. Different letters above each bar indicate significant differences: $P < 0.05$, whereas bars with the same letter indicates non-significant differences.

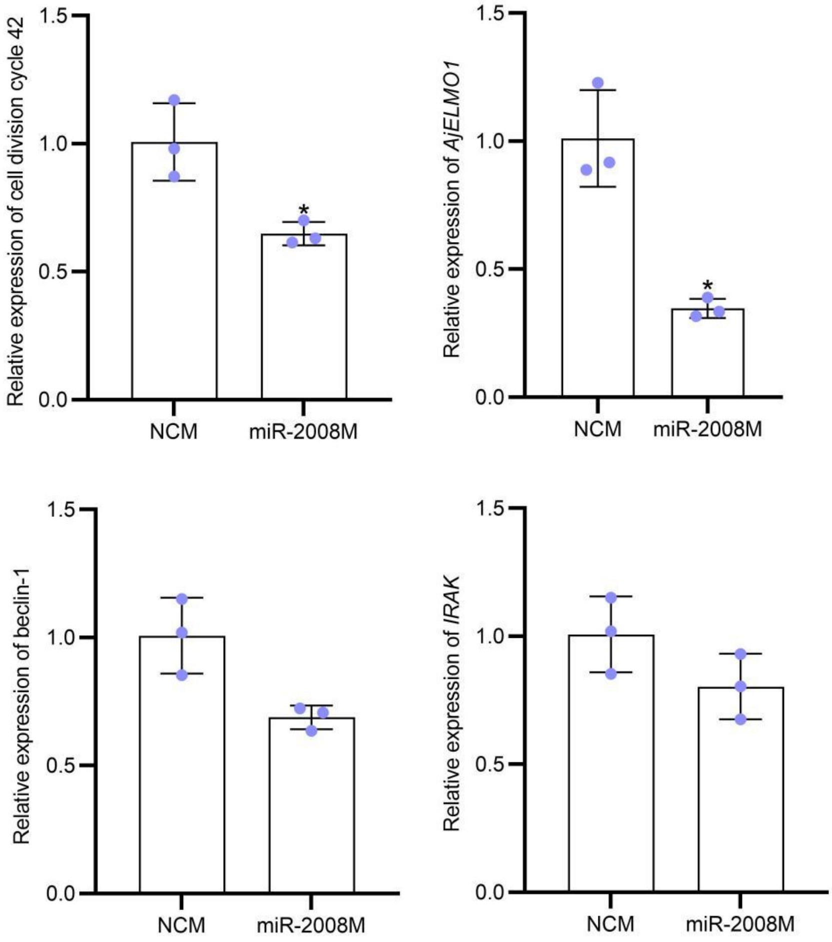

Supplementary Figure 3 The relative abundance level of the four genes (cell division cycle 42, beclin-1, interleukin-1 receptor-associated kinase, and *AjELMO1*) after transfection with modified miR-2008 mimics *in vivo* by qPCR. All data represented the mean ± SD from three independent triplicated experiments. *$P < 0.05$, **$P < 0.01$.

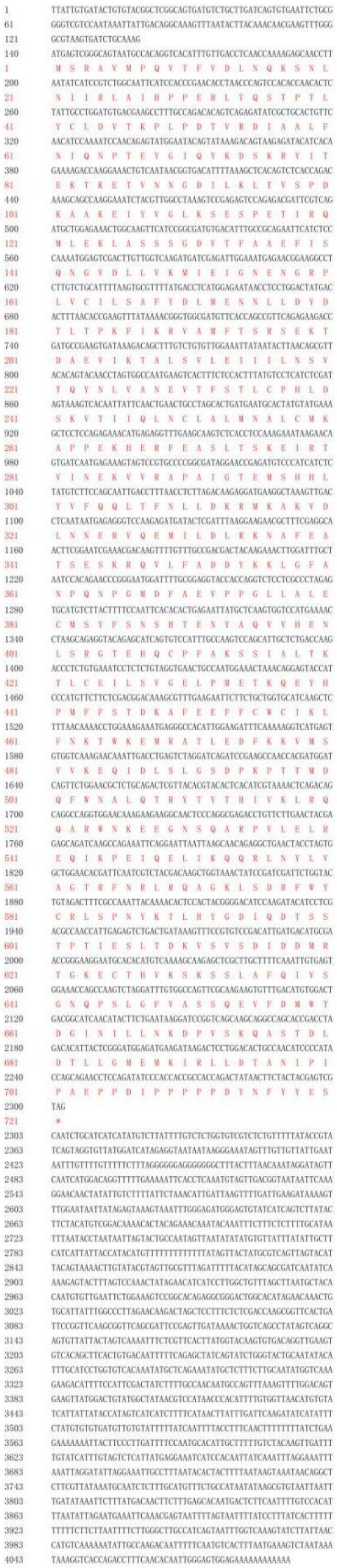

Supplementary Figure 4 The cDNAs and deduced amino acid sequences of *AjELMO1*.

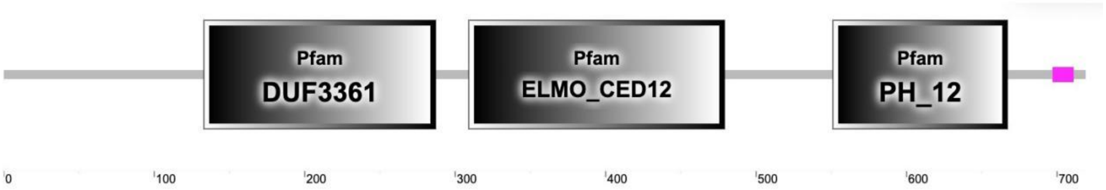

Supplementary Figure 5 The domain architecture of AjELMO1 was predicted by SMART.

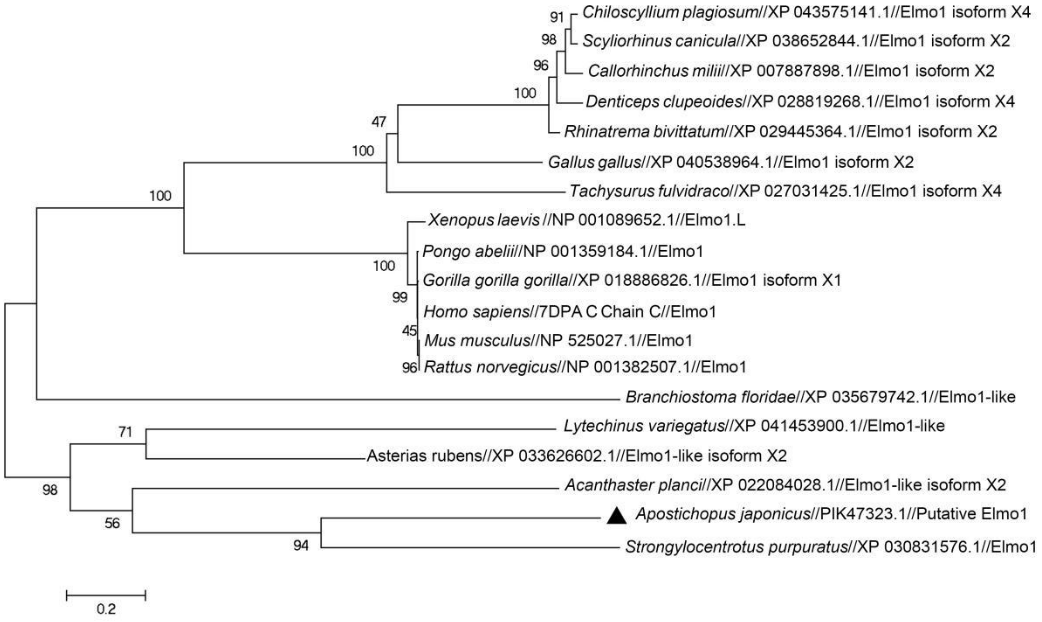

Supplementary Figure 6 The phylogenic tree was constructed using MEGA 7.0 to analyze the evolutionary relationship between AjELMO1 and other ELMO1 family members.

Figure S6

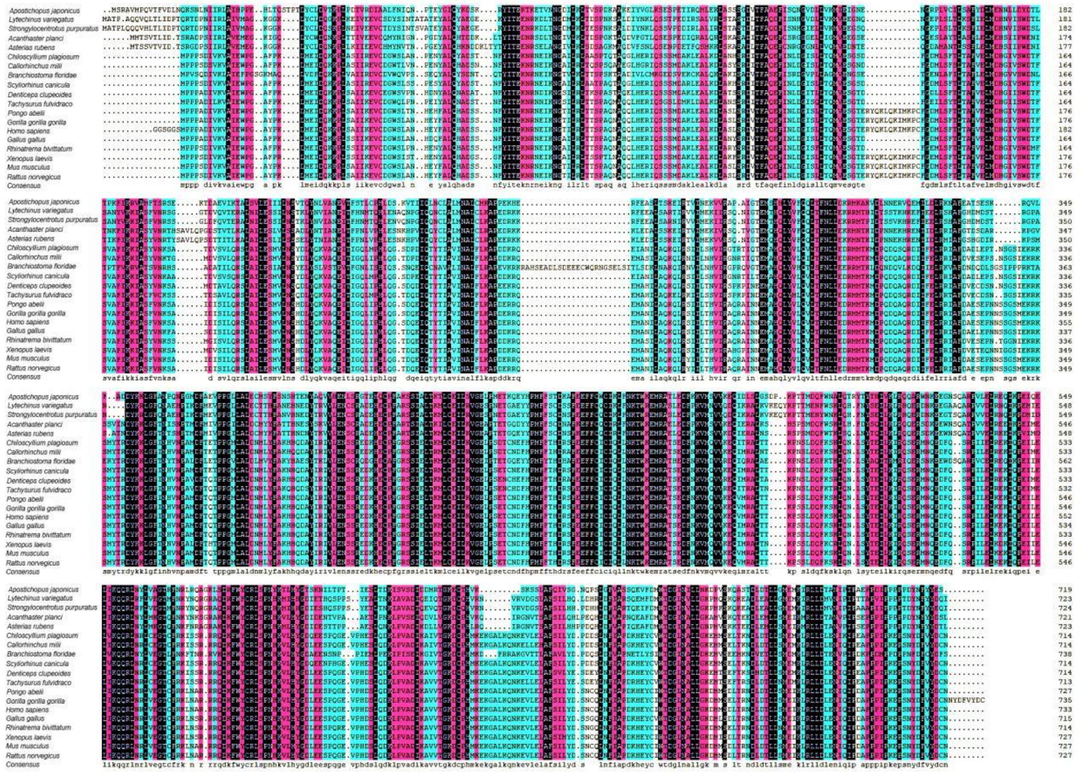

Supplementary Figure 7 Multiple sequence alignments of AjELMO1 and other ELMO1 proteins by using the DNAMAN software.

Fig. 5e

In vivo AjELMO1 Ajβ-actin

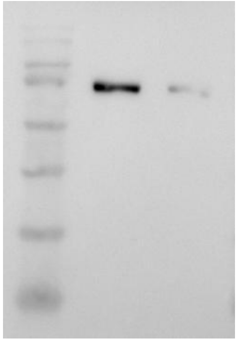 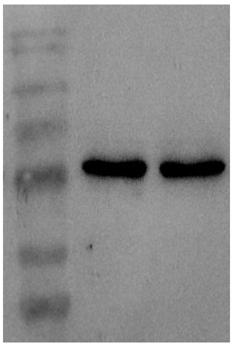

In vitro AjELMO1 Ajβ-actin

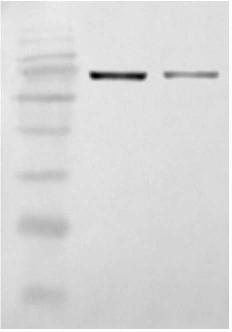 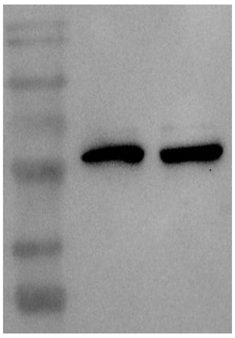

Fig. 5f

In vivo AjELMO1 Ajβ-actin

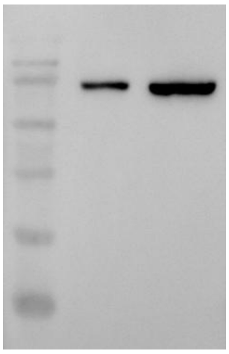 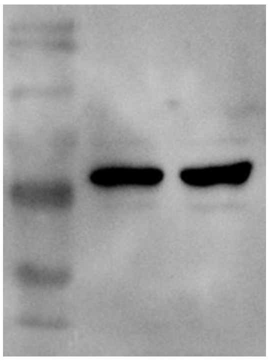

In vitro AjElmo1 Ajβ-actin

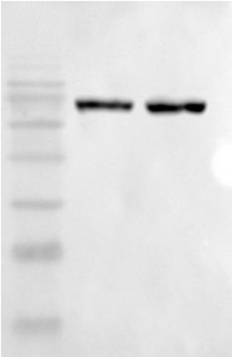 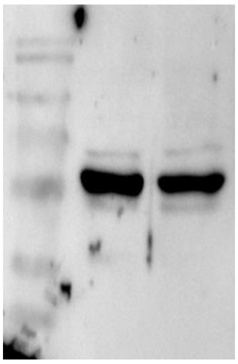

Fig. 6b

In vivo AjELMO1 Ajβ-actin

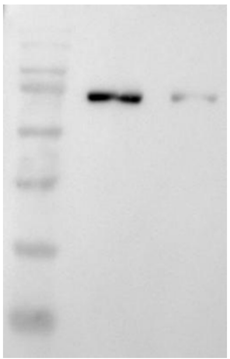

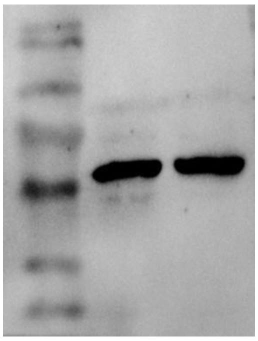

In vitro AjELMO1 Ajβ-actin

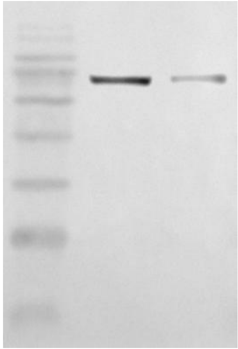

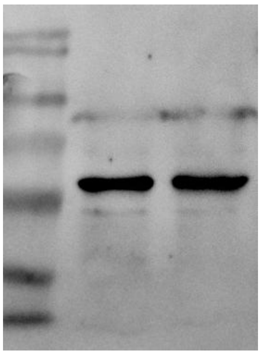

Fig. 7a

In vivo AjELMO1 Ajβ-actin

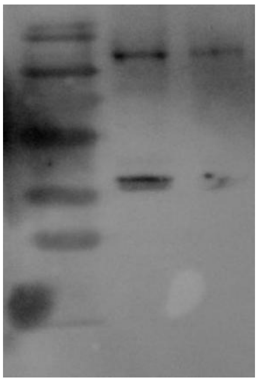

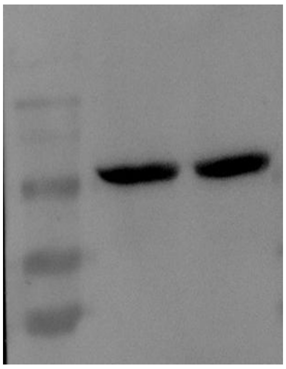

In vitro AjELMO1 Ajβ-actin

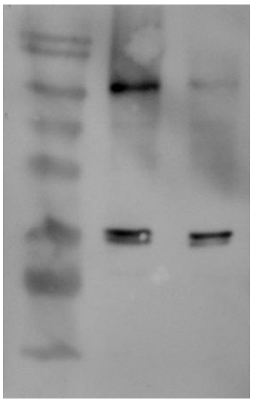

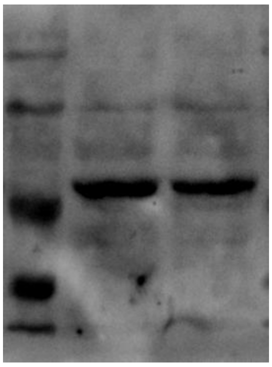

Fig. 7b

In vivo AjELMO1 Ajβ-actin

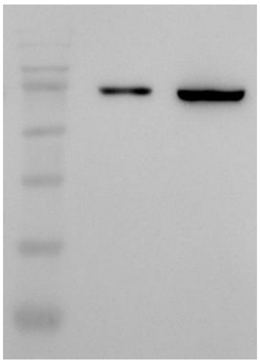

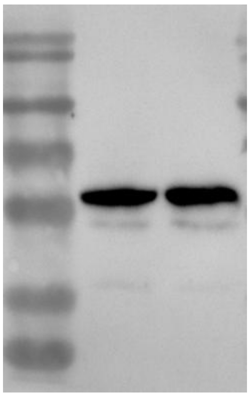

In vitro AjELMO1 Ajβ-actin

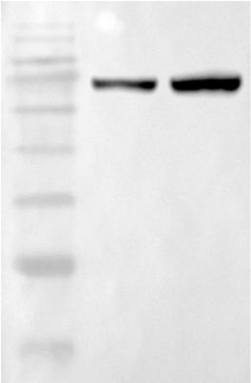

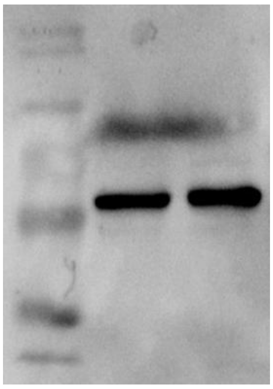

Supplementary Figure 8 Western blot validated that the effect of AjELMO1 and Ajβ-actin proteins caused by modified miR-2008 mimics and inhibitors (Fig. 5e, Fig.5f) or AjELMO1 siRNA transfection (Fig. 6b) or circ432 siRNA transfection (Fig. 7a) or circ432 siRNA transfection and modified miR-2008 inhibitor (Fig. 7b) *in vivo* and *in vitro*.

Supplementary Note 1: MIQE support information and supplementary methods and tables

1. EXPERIMENTAL DESIGN AND SAMPLES

Varanasi specific to circ432 was designed and synthesized by GenePharma (Shanghai, China) (Table 1). Another siRNA (negative control, NC) that was not specific for any unigenes in the *A. japonicus* transcriptome served as a negative control. These siRNAs were then dissolved in RNase-free water to generate 20 μM working solutions. For RNA interference, 10 μL circ432 siRNA and an equal volume of transfection reagent were mixed with 80 μL of phosphate-buffered saline (PBS) to prepare the transfection solution. Each sea cucumber (120 ± 5 g) was injected with 100 μL of the transfection solution described above by tentacle injection. The control group was injected with NC siRNA under the same conditions. Similar method was used for the *AjELMO1* knockdown assay. At 48 h posttransfection, control and treated coelomocytes were harvested to assess the silencing efficiency. The treated and negative control groups were set up in triplicate.

2. NUCLEIC ACID EXTRACTION

The total RNA was extracted from the coelomocytes of *A. japonicus* by using the RNAiso plus reagent (TaKaRa, Ootsu, Japan). The total RNA was extracted from the coelomocytes of *A. japonicus* by using the RNAiso plus reagent and treated with the RNase-free DNase I to remove the genomic DNA according to the manufacturer’s protocol. Polysaccharides, membranes, and unlysed cells are eliminated by centrifugation (12,000 g, 10 min, 4℃). At this step, the supernatant is immediately treated according to the supplier protocol. Briefly, add 200 μL of chloroform, incubate at room temperature for 2 min, and centrifuge (12,000 g, 15 min, 4℃). The supernatant (600 μL cemented with 500 μL of 2-propanol and centrifuged (12,000 g, 10 min, 4℃) after a 10-min incubation at room temperature. The RNA pellet is washed (1 mL of 70%(v/v)

ethanol and centrifugation at 8000 g for 10 min at 4℃), dried at room temperature for 30 min, and resuspended in 20 μL of Milli-Q (Millipore, Bedford, MA)-treated sterile water.

This table details the validation of each RNA used to generate data for this study.

**Sample ID**	**Nucleic Acid**	**Unit**	**A260 (Abs)**	**A280 (Abs)**	**A260/A280**	**A260/A280=1.8~2.0**	**Sample Type**
si-NC1 (*in vivo*)	660.6	ng/μL	16.514	8.450	1.95	Yes, standards-compliant	RNA
si-NC2 (*in vivo*)	633.6	ng/μL	15.840	7.946	1.99	Yes, standards-compliant	RNA
si-NC3 (*in vivo*)	536.3	ng/μL	13.409	6.698	2.00	Yes, standards-compliant	RNA
si-circ432-1 (*in vivo*)	2897.8	ng/μL	72.445	36.447	1.99	Yes, standards-compliant	RNA
si-circ432-2 (*in vivo*)	2269.3	ng/μL	56.734	28.504	1.99	Yes, standards-compliant	RNA
si-circ432-3 (*in vivo*)	3644.3	ng/μL	91.108	45.613	2.00	Yes, standards-compliant	RNA
si-*AjELMO1*-1 (*in vivo*)	1380.7	ng/μL	34.518	17.301	2.00	Yes, standards-compliant	RNA

si-*AjELMO1*-2 (*in vivo*)	825.8	ng/μL	20.644	10.330	2.00	Yes, standards-compliant	RNA
si-*AjELMO1*-3 (*in vivo*)	659.0	ng/μL	16.475	8.708	1.89	Yes, standards-compliant	RNA
si-NC1 (*in vitro*)	306.1	ng/μL	7.652	3.920	1.95	Yes, standards-compliant	RNA
si-NC2 (*in vitro*)	250.6	ng/μL	6.266	3.172	1.98	Yes, standards-compliant	RNA
si-NC3 (*in vitro*)	355.9	ng/μL	8.898	4.570	1.95	Yes, standards-compliant	RNA
si-circ432-1 (*in vitro*)	385.3	ng/μL	9.633	4.833	1.99	Yes, standards-compliant	RNA
si-circ432-2 (*in vitro*)	311.7	ng/μL	7.792	3.963	1.97	Yes, standards-compliant	RNA
si-circ432-3 (*in vitro*)	204.6	ng/μL	5.114	2.689	1.90	Yes, standards-compliant	RNA
si-*AjELMO1*-1 (*in vitro*)	180.1	ng/μL	4.503	2.358	1.91	Yes, standards-compliant	RNA
si-*AjELMO1*-2 (*in vitro*)	176.3	ng/μL	4.407	2.226	1.98	Yes, standards-complia	RNA

						nt	
si-*AjELMO1* -3 (*in vitro*)	190.9	ng/μL	4.77 2	2.512	1.90	Yes, standards-complia nt	RNA

3. REVERSE TRANSCRIPTION

Genomic DNA removal reaction

Reagent	Volume of use
5 × gDNA Eraser Buffer	2 μL
gDNA Eraser	1 μL
Total RNA	1 μg
RNase Free H_2O	Up to 10 μL

↓

42℃ 2 min

↓

Reverse Transcription

(TB Green qPCR method)

Reagent	volume of use
The reaction solution from the previous step	10 μL
PrimerScript RT Enzyme MIX 1	1 μL
RT Primer	4 μL
5 × PrimerScript Buffer 2 (for Real Time)	4 μL
RNase Free H_2O	1 μL
Total	20 μL

↓

37℃ 15min

↓

85℃ 5sec

4. qPCR TARGET INFORMATION and qPCR OLIGONUCLEOTIDES

Assay ID	Oligo ID	Oligonucleotide sequence and modification (5’ to 3’)	In silico verificcation	Amplicon length
circ432	Shown in Table 1	Shown in Table 1	Yes; specific for target	253 bp
AjELMO1	Shown in Table 1	Shown in Table 1	Yes; specific for target	212 bp
Ajβ-actin	Shown in Table 1	Shown in Table 1	Yes; specific for target	154 bp

5. qPCR PROTOCOL

The transcripts of genes were analyzed via quantitative real-time PCR (qRT-PCR) on a Applied Biosystem 7500 real-time PCR system. According to the manufacturer's protocol, total RNA were extracted with the TRIzol reagent (TaKaRa, Ootsu, Japan), and cDNA was prepared using PrimeScript™ RT reagent with gDNA Eraser Kit (TaKaRa, Ootsu, Japan). Amplification was conducted in a 20 μL reaction volume containing 8 μL of 1:50 diluted cDNA, 0.8 μL of each primer (listed in Table 1), 10 μL of SYBR Green, and 0.4 μL of ROX. The reaction mixtures were incubated for 2 min at 95℃, followed by 40 cycles of 15 s at 95℃, 15 s at 60℃, and 20 s at 72℃ followed by a melting curve. The baseline was automatically set by the software to maintain consistency. The relative expression levels were calculated using the $2^{-\Delta\Delta CT}$ method with β-actin for normalization. Each PCR trial was run in triplicate parallel reactions and repeated three times. The primer efficiency was checked. A significant difference in expression relative to expression in the control group at each time point is indicated using an asterisk for $p < 0.05$ and two asterisks for $p < 0.01$.

6. qPCR VALIDATION

This table details the validation for each Primer used to generate the figures in this study.

Stand Curve of Ajβ-actin

Slope: -3.3345 Intercept: 31.775 y=--3.3345x + 31.775 Ajβ-actin PCR Effidency: 99%

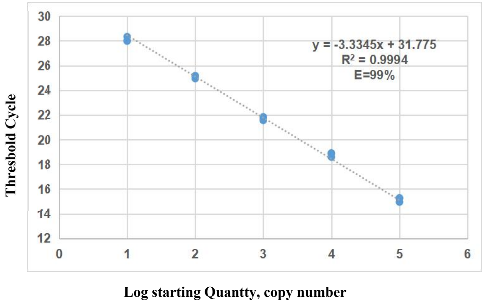

Amplification Plot of Ajβ-actin

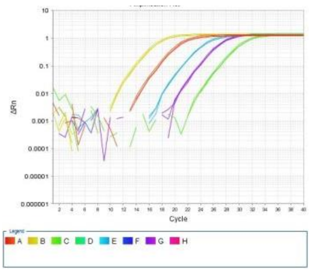

Melt Curve of Ajβ-actin

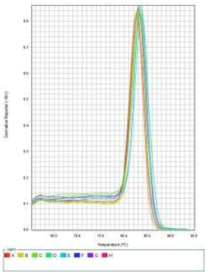

Stand Curve of *AjELMO1*

Slope: -3.3193 Intercept: 31.566 y=-3.3193x + 31.556 AjElmo1 PCR Effidency: 100%

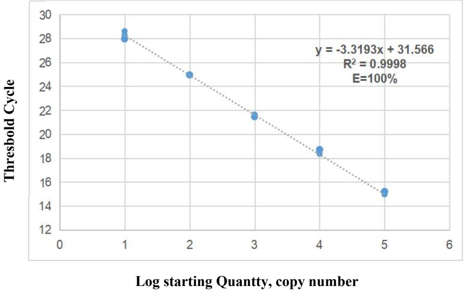

Amplification Plot of *AjELMO1*

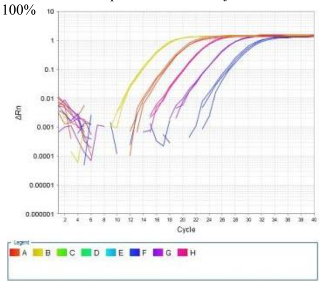

Melt Curve of *AjELMO1*

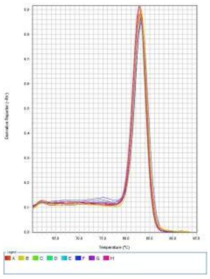

Stand Curve of circ432

Slope: -3.2807 Intercept: 36.698 y=-3.2807x + 36.698 circ432 PCR Effidency: 102%

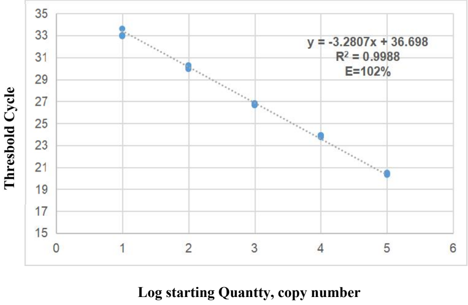

Amplification Plot of circ432

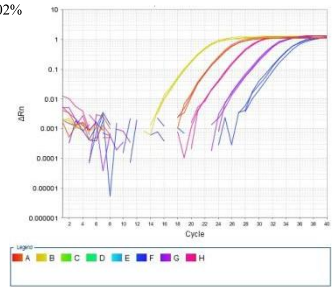

Melt Curve of circ432

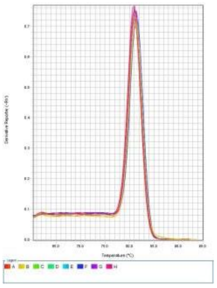

7. DATA ANALYSIS

The files were imported into Excel to calculate ΔC_T, $\Delta\Delta C_T$, Power, STDEV and T-test Calculated as follows:

$\Delta C_T = C_{T\ target} - C_{T\ reference}$

The standard deviation of ΔC_T (ΔC_T SD)

$=\sqrt{(\text{SD of } C_{T\ target})^2 + (\text{SD of } C_{T\ reference})^2)}$

$\Delta\Delta C_T = \Delta C_T$ sample $- \Delta C_T$ calibrator

Fold change (indole/DMSO, normalized by proC) = $2^{-\Delta\Delta CT}$

8. All qPCR data in this experiment are shown in the following table

Table 1. The cycling numbers and fold change of circ432 in different tissues. （**Fig 2a**）

Gene	Ajβ-actin (reference gene)					circ432				
Treatment	tentacles	muscles	respiratory trees	coelomocytes	intestines	tentacles	muscles	respiratory trees	coelomocytes	intestines
C_T 1	19.56	20.04	18.79	21.08	24.31	24.73	23.67	23.11	22.81	24.31
C_T 2	19.18	19.76	18.48	20.89	23.46	24.13	23.01	23.01	22.60	23.46
C_T 3	19.52	19.94	18.06	21.03	22.81	24.90	23.28	23.17	22.91	23.66
C_T Mean	19.42 ± 0.209	19.91 ± 0.142	18.44 ± 0.366	21.00 ± 0.098	21.15 ± 0.156	24.59 ± 0.405	23.32 ± 0.332	23.10 ± 0.081	22.77 ± 0.158	23.81 ± 0.444
ΔC_T						5.17 ± 0.215	3.41 ± 0.199	4.65 ± 0.409	1.77 ± 0.093	2.66 ± 0.357
$\Delta\Delta C_T$							-1.76	-0.52	-3.4	2.51
Fold change							3.387	1.434	10.556	5.696

Table 2. The cycling numbers and fold change of circ432 in *V. s* treated different times. （**Fig 2b**）

Gene	Ajβ-actin (reference gene)					circ432				
Treatment	0 h	12 h	24 h	48 h	72 h	0 h	12 h	24 h	48 h	72 h
C_T 1	19.00	19.07	20.35	18.95	18.90	27.89	25.49	25.93	25.02	26.84
C_T 2	18.81	18.93	19.98	18.74	18.62	27.95	27.55	26.30	24.91	26.71
C_T 3	19.13	19.21	20.60	19.04	18.55	27.43	26.62	26.55	24.58	26.46
C_T Mean	18.98 ± 0.161	19.07 ± 0.140	20.31 ± 0.312	18.91 ± 0.154	18.69 ± 0.185	27.75 ± 0.286	26.55 ± 1.033	26.26 ± 0.310	24.84 ± 0.230	26.67 ± 0.191
ΔC_T						8.78 ± 0.431	7.48 ± 1.102	5.95 ± 0.370	5.93 ± 0.339	7.98 ± 0.096
$\Delta\Delta C_T$							-1.3	-2.83	-2.85	-0.8
Fold change							2.462	7.111	7.210	1.74

Table 3. The cycling numbers and fold change of circ432 in LPS treated different times. （**Fig 2b**）

Gene	Ajβ-actin (reference gene)					circ432				
Treatment	0 h	3 h	6 h	12 h	24 h	0 h	3 h	6 h	12 h	24 h
C_T 1	15.02	15.80	17.04	16.80	15.92	25.96	25.48	26.37	26.45	25.88
C_T 2	15.13	15.74	16.90	16.95	16.13	25.63	25.69	26.43	26.42	25.97
C_T 3	15.33	16.13	17.03	16.89	16.04	25.55	25.48	26.11	26.16	25.97
C_T Mean	15.16 ± 0.157	15.89 ± 0.210	16.99 ± 0.078	16.88 ± 0.075	16.03 ± 0.105	25.71 ± 0.215	25.55 ± 0.118	26.30 ± 0.170	26.34 ± 0.158	25.94 ± 0.053
ΔC_T						10.55 ± 0.363	9.66 ± 0.300	9.31 ± 0.225	9.46 ± 0.190	9.91 ± 0.062
$\Delta\Delta C_T$							-0.89	-1.24	-1.09	-0.64
Fold change							1.853	2.36	2.13	1.56

Table 4. The cycling numbers and fold change of circ432 in si-circ432-1 *in vivo*. （**Fig 2c**）

Gene	Ajβ-actin (reference gene)		circ432	
Treatment	si-NC	si-circ432-1	si-NC	si-circ432-1
C_T 1	18.94	18.12	26.71	26.83
C_T 2	18.89	17.92	26.84	26.63
C_T 3	18.93	17.98	26.69	26.75
C_T Mean	18.92 ± 0.026	18.01 ± 0.103	26.75 ± 0.081	26.74 ± 0.101
ΔC_T			7.83 ± 0.107	8.73 ± 0.035
$\Delta\Delta C_T$			0.9	
Fold change			0.536	

Table 5. The cycling numbers and fold change of circ432 in si-circ432-1 *in vitro*. （**Fig 2c**）

Gene	Ajβ-actin (reference gene)		circ432	
Treatment	si-NC	si-circ432-1	si-NC	si-circ432-1
C_T 1	18.73	17.93	27.87	28.12
C_T 2	18.65	17.92	27.60	27.95
C_T 3	18.73	17.98	27.69	27.97
C_T Mean	18.70 ± 0.046	18.00 ± 0.032	27.72 ± 0.137	28.01 ± 0.093
ΔC_T			9.02 ± 0.107	10.07 ± 0.106
$\Delta\Delta C_T$			1.05	
Fold change			0.483	

Table 6. The cycling numbers and fold change of circ432 in si-circ432-2 *in vivo*. （**Fig 2c**）

Gene	Ajβ-actin (reference gene)		circ432	
Treatment	si-NC	si-circ432-2	si-NC	si-circ432-2
C_T 1	18.73	18.72	23.82	24.39
C_T 2	18.65	18.66	23.85	24.28
C_T 3	18.73	18.60	23.69	24.01
C_T Mean	18.70 ± 0.043	18.66 ± 0.058	23.79 ± 0.086	24.23 ± 0.196
ΔC_T			5.08 ± 0.118	5.56 ± 0.139
$\Delta\Delta C_T$			0.48	
Fold change			0.717	

Table 7. The cycling numbers and fold change of circ432 in si-circ432-2 *in vitro*. （**Fig 2c**）

Gene	Ajβ-actin (reference gene)		circ432	
Treatment	si-NC	si-circ432-2	si-NC	si-circ432-2
C_T 1	18.53	18.42	26.40	27.34
C_T 2	18.35	18.35	26.29	27.04
C_T 3	17.98	18.31	26.28	26.90
C_T Mean	18.29 ± 0.280	18.36 ± 0.055	26.32 ± 0.068	27.09 ± 0.223
ΔC_T			8.03 ± 0.229	8.73 ± 0.168
$\Delta\Delta C_T$			0.7	
Fold change			0.616	

Table 8. The cycling numbers and fold change of miR-2008 in miR-2008M *in vivo*. （**Fig 4a**)

Gene	RNU6B (reference gene)		miR-2008	
Treatment	NCM	miR-2008M	NCM	miR-2008M
C_T 1	31.66	35.95	33.89	31.18
C_T 2	32.27	35.57	34.50	31.03
C_T 3	32.27	34.69	34.24	30.99
C_T Mean	32.07 ± 0.350	35.40 ± 0.647	34.21 ± 0.309	31.067 ± 0.104
ΔC_T			2.14 ± 0.147	-4.33 ± 0.561
$\Delta\Delta C_T$			-6.47	
Fold change			88.647	

Table 9. The cycling numbers and fold change of miR-2008 in miR-2008M *in vitro*. （**Fig 4a**)

Gene	RNU6B (reference gene)		miR-2008	
Treatment	NCM	miR-2008M	NCM	miR-2008M
C_T 1	29.97	34.43	33.82	29.31
C_T 2	29.86	35.00	33.53	29.47
C_T 3	30.70	35.00	35.00	29.46
C_T Mean	30.18 ± 0.457	34.81 ± 0.329	34.11 ± 0.779	29.41 ± 0.090
ΔC_T			3.94 ± 0.324	-5.40 ± 0.240
$\Delta\Delta C_T$			-9.34	
Fold change			648.067	

Table 10. The cycling numbers and fold change of miR-2008 in miR-2008I *in vivo*. （**Fig 4a**)

Gene	RNU6B (reference gene)		miR-2008	
Treatment	NCI	miR-2008I	NCI	miR-2008I
C_T 1	31.66	33.62	33.89	36.71
C_T 2	32.27	33.96	34.50	36.98
C_T 3	32.27	35.45	34.24	38.47
C_T Mean	32.07 ± 0.350	34.34 ± 0.973	34.21 ± 0.309	37.39 ± 0.949
ΔC_T			2.14 ± 0.147	3.04 ± 0.041
$\Delta\Delta C_T$			0.9	
Fold change			0.536	

Table 10. The cycling numbers and fold change of miR-2008 in miR-2008I *in vitro*. （**Fig 4a**)

Gene	RNU6B (reference gene)		miR-2008	
Treatment	NCI	miR-2008I	NCI	miR-2008I
C_T 1	35.12	30.43	34.34	33.14
C_T 2	34.92	30.44	35.00	32.82
C_T 3	34.96	30.70	35.00	32.46
C_T Mean	35.00 ± 0.106	30.52 ± 0.153	34.78 ± 0.381	32.81 ± 0.340
ΔC_T			-0.22 ± 0.381	2.28 ± 0.482
$\Delta\Delta C_T$			2.5	
Fold change			0.177	

Table 11. The cycling numbers and fold change of *AjELMO1* in *V. s* treated different times. （**Fig 6a**)

Gene	Ajβ-actin (reference gene)					*AjELMO1*				
Treatment	0 h	12 h	24 h	48 h	72 h	0 h	12 h	24 h	48 h	72 h
C_T 1	22.27	19.47	19.76	18.27	18.15	33.36	27.94	27.77	27.75	27.37
C_T 2	24.00	18.93	19.89	19.20	18.37	34.64	27.22	28.12	27.86	28.25
C_T 3	23.01	18.76	19.07	17.13	17.26	34.23	27.17	27.40	26.70	26.75
C_T Mean	23.09 ± 0.865	19.05 ± 0.369	19.57 ± 0.441	18.20 ± 1.037	17.93± 0.588	34.08 ± 0.652	27.44 ± 0.431	27.76 ± 0.364	27.44 ± 0.642	27.46 ± 0.751
ΔC_T						10.98 ± 0.302	8.39 ± 0.092	8.19 ± 0.159	9.24 ± 0.502	9.52 ± 0.332
$\Delta\Delta C_T$							-2.59	-2.79	-1.74	-1.46
Fold change							6.021	6.916	3.340	2.751

Table 12. The cycling numbers and fold change of *AjElLMO1* in LPS treated different times. （**Fig 6a**)

Gene	Ajβ-actin (reference gene)					*AjELMO1*				
Treatment	0 h	3 h	6 h	12 h	24 h	0 h	3 h	6 h	12 h	24 h
C_T 1	17.72	18.06	19.30	18.52	17.91	25.43	25.14	25.76	25.49	25.48
C_T 2	17.65	18.05	19.22	18.25	17.95	25.31	25.13	25.64	25.48	25.50
C_T 3	17.62	17.91	19.17	18.19	17.30	25.14	25.08	25.73	25.44	25.33
C_T Mean	17.66 ± 0.051	18.01 ± 0.084	19.23 ± 0.066	18.32 ± 0.176	17.72 ± 0.364	25.29 ± 0.146	25.12 ± 0.032	25.71 ± 0.062	25.47 ± 0.026	25.44 ± 0.093
ΔC_T						7.63 ± 0.098	7.11 ± 0.052	6.48 ± 0.072	7.15 ± 0.156	7.72 ± 0.272
$\Delta\Delta C_T$							-0.52	-1.15	-0.48	-0.09
Fold change							1.434	2.219	1.395	1.064

Table 13. The cycling numbers and fold change of *AjELMO1* in si-*AjELMO1 in vivo*. （**Fig 6b**）

Gene	Ajβ-actin (reference gene)		*AjELMO1*	
Treatment	si-NC	si-*AjELMO1*	si-NC	si-*AjELMO1*
C_T 1	18.90	19.61	26.32	27.48
C_T 2	18.89	19.60	26.20	27.63
C_T 3	18.84	19.58	26.22	27.56
C_T Mean	18.88 ± 0.032	19.60 ± 0.015	26.25 ± 0.064	27.56 ± 0.075
ΔC_T			7.37 ± 0.056	7.96 ± 0.082
$\Delta\Delta C_T$			0.59	
Fold change			0.664	

Table 13. The cycling numbers and fold change of *AjELMO1* in si-*AjELMO1 in vitro*. （**Fig 6b**）

Gene	Ajβ-actin (reference gene)		*AjELMO1*	
Treatment	si-NC	si-*AjELMO1*	si-NC	si-*AjELMO1*
C_T 1	19.61	19.28	26.67	26.94
C_T 2	19.47	19.18	26.26	26.70
C_T 3	19.26	19.02	26.12	26.48
C_T Mean	19.45 ± 0.176	19.16 ± 0.131	26.35 ± 0.286	26.71 ± 0.230
ΔC_T			6.90 ± 0.140	7.55 ± 0.103
$\Delta\Delta C_T$			0.65	
Fold change			0.637	

Table 14. The cycling numbers and fold change of *AjELMO1* in si-circ432 *in vivo*. （**Fig 7a**）

Gene	Ajβ-actin (reference gene)		*AjELMO1*	
Treatment	si-NC	si-circ432	si-NC	si-circ432
C_T 1	18.03	17.95	24.92	25.82
C_T 2	17.82	17.89	24.96	25.92
C_T 3	17.68	17.98	24.45	25.82
C_T Mean	17.84 ± 0.177	17.94 ± 0.044	24.78 ± 0.282	25.85 ± 0.058
ΔC_T			6.94 ± 0.188	7.91 ± 0.099
$\Delta\Delta C_T$			0.97	
Fold change			0.511	

Table 15. The cycling numbers and fold change of miR-2008 in si-circ432 *in*

vivo. （**Fig 7a**)

Gene	RNU6B (reference gene)		miR-2008	
Treatment	si-NC	si-circ432	si-NC	si-circ432
C_T 1	27.34	29.01	30.77	32.57
C_T 2	26.93	28.97	30.56	32.48
C_T 3	27.44	29.50	30.60	32.47
C_T Mean	27.24 ± 0.274	29.17 ± 0.298	30.64 ± 0.113	32.51 ± 0.052
ΔC_T			3.40 ± 0.240	3.34 ± 0.329
$\Delta\Delta C_T$			-0.06	
Fold change			1.042	

Table 16. The cycling numbers and fold change of *AjELMO1* in si-circ432 *in vitro*. （**Fig 7a**)

Gene	Ajβ-actin (reference gene)		*AjELMO1*	
Treatment	si-NC	si-circ432	si-NC	si-circ432
C_T 1	19.12	18.31	27.08	27.45
C_T 2	18.84	17.98	26.99	27.53
C_T 3	18.71	17.93	26.88	27.50
C_T Mean	18.89 ± 0.209	18.07 ± 0.203	26.98 ± 0.097	27.50 ± 0.041
ΔC_T			8.09 ± 0.117	9.42 ± 0.239
$\Delta\Delta C_T$			1.33	
Fold change			0.398	

Table 17. The cycling numbers and fold change of miR-2008 in si-circ432 *in vitro*. （**Fig 7a**)

Gene	RNU6B (reference gene)		miR-2008	
Treatment	si-NC	si-circ432	si-NC	si-circ432
C_T 1	24.71	28.32	31.19	34.93
C_T 2	24.58	28.23	30.99	34.28
C_T 3	24.50	28.08	30.92	34.25
C_T Mean	24.60 ± 0.104	28.21 ± 0.121	31.03 ± 0.139	34.49 ± 0.386
ΔC_T			6.44 ± 0.039	6.28 ± 0.297
$\Delta\Delta C_T$			-0.16	
Fold change			1.117	

Table 18. The cycling numbers and fold change of *AjELMO1* in si-circ432 and miR-2008I *in vivo*. （**Fig 7a**)

Gene	Ajβ-actin (reference gene)		*AjELMO1*	
Treatment	si-NC	si-circ432 & miR-2008I	si-NC	si-circ432 & miR-2008I
C_T 1	16.49	16.96	25.31	25.22
C_T 2	16.17	16.99	25.10	25.29
C_T 3	15.92	17.00	24.92	25.43
C_T Mean	16.19 ± 0.285	16.98 ± 0.019	25.11 ± 0.200	25.31 ± 0.106
ΔC_T			8.92 ± 0.086	8.33 ± 0.090
$\Delta\Delta C_T$			-0.59	
Fold change			1.505	

Table 19. The cycling numbers and fold change of *AjELMO1* in si-circ432 and miR-2008I *in vitro*. （**Fig 7a**)

Gene	Ajβ-actin (reference gene)		*AjELMO1*	
Treatment	si-NC	si-circ432 & miR-2008I	si-NC	si-circ432 & miR-2008I
C_T 1	16.07	17.44	25.17	25.73
C_T 2	16.11	17.39	25.14	25.59
C_T 3	16.11	17.19	25.16	25.54
C_T Mean	16.09 ± 0.023	17.34 ± 0.134	25.16 ± 0.015	25.62 ± 0.099
ΔC_T			9.06 ± 0.035	8.28 ± 0.074
$\Delta\Delta C_T$			-0.78	
Fold change			1.717	

Supplementary Note 2: Selection of reference genes for qRT-PCR analysis of gene expression in *Apostichopus japonicus* during *Vibrio splendidus* infection

1. Primer design

In this study, ten candidate reference genes based on the frequently used housekeeping genes and genes that showed stable expression in our acquired RNA-seq transcriptome data (Zhang et al., 2014, Fish Shellfish Immunol. 38, 383–388), including Ajβ-actin, RPS19, RPS18, GAPD, NADH, TUBA, TUBB, NDUFA13, RPL18A, and EF1α. These ten potential reference genes were also selected from different functional categories, which encode a variety of proteins with different functions, including cytoskeletal proteins, tubulin, proteins that play a role in the electronic transport system of the respiratory chain, and three ribosomal subunit proteins. Primers (**Table 1**) were designed for each gene using Primer 5.0 Software.

Table 1 Candidate reference genes and their primer sequences for qRT-PCR.

Symbol	Gene name	Function	Primer sequences (5'-3')	Product size (bp)
Ajβ-actin	β-actin	Cytoskeletal protein	F:CCATTCAACCCTAAAGCCAACA<br>R: ACACACCGTCTCCTGAGTCCAT	154 bp
RPS9	40S ribosomal protein S9	40S ribosomal subunit	F: GTCAACATCCCATCCTTT<br>R: TTCCTCTTCTTCTCCTCC	150 bp
GAPD	glyceraldehyde 3-phosphate dehydrogenase	Oxidoreductase in glycolysis and gluconeogenesis	F: GCTGCGAAGGCTGTTGGA<br>R: ACTGCTGGCTGCTTTTTT	168 bp
NADH	NADH dehydrogenase	Electron transport in the respiratory chain	F: GTCCTACGACCCAATCTGGA<br>R: ATGAGCCTTGGTTACGTTGG	196 bp
TUBA	α-Tubulin	Microtubule protein	F: TGGAAGATGAGGAACCCTTG<br>R: GGAAAGGAGCAAATCGATCA	86 bp
NDUFA13	NADH dehydrogenase [ubiquinone] 1 α	Electron transport in the respiratory chain	F:GACATTCTGAAGCAACTGCAAC<br>R: AACATTGCCTCATTCTGTTCCT	170 bp
RPL18A	60S ribosomal protein L18a	60S ribosomal subunit	F: CAGAGGGAACTTCAGCTTGC<br>R: TACCCAGTGCTACCGTGACA	144 bp
TUBB	β-Tubulin	Microtubule protein	F: GAAAGCCTTACGACGGAACA<br>R: CACCACGTGGACTCAAAATG	113 bp
EF1α	elongation factor-1 alpha	Essential component of the eukaryotic translational apparatus	F: GTTCGAAACCAAAAAGTA<br>R: GTGTAAGCCAATAAGCAT	188 bp
RPS18	40S ribosomal protein S18	40S ribosomal subunit	F: GACGGCAAGTTCAACCAGAT<br>R: GTGCTGACCTCTGACACGAA	132 bp

2. qPCR Validation

We confirmed the specificity of PCR by melting curve analysis to check that only one single peak was obtained in each determination. PCR efficiency analysis showed that the E value of these ten candidate reference genes ranged from 96% to 107%, and the R^2 range was greater than 0.99 (**Fig. 1**).

Stand Curve of Ajβ-actin

Slope: -3.3345 Intercept: 31.775 y=--3.3345x + 31.775 Ajβ-actin PCR Effidency: 99%

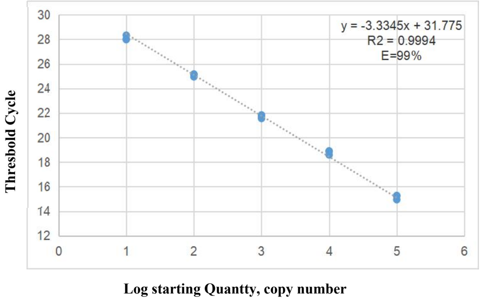

Amplification Plot of Ajβ-actin

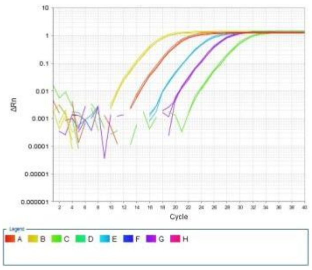

Melt Curve of Ajβ-actin

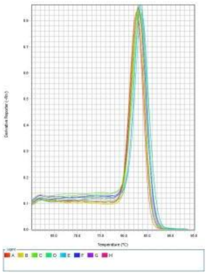

Stand Curve of RPS9

Slope: -3.1694 Intercept: 32.963 y=--3.1694x + 32.963 RPS9 PCR Effidency: 106%

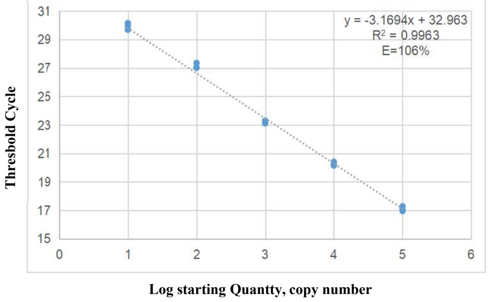

Amplification Plot of RPS9

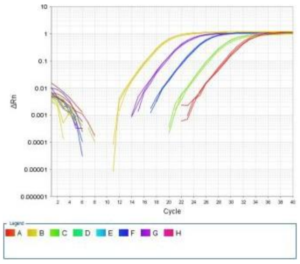

Melt Curve of RPS9

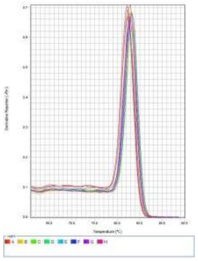

Stand Curve of GAPD

Slope: -3.1287 Intercept: 37.682 y=--3.1287x + 37.682 GAPD PCR Effidency: 108%

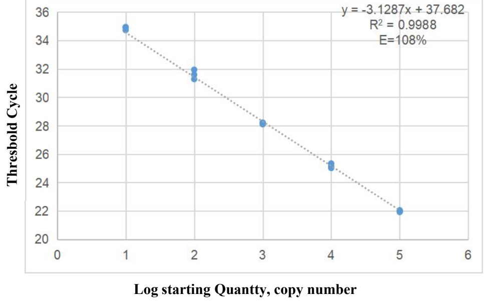

Amplification Plot of GAPD

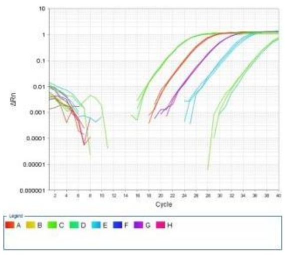

Melt Curve of GAPD

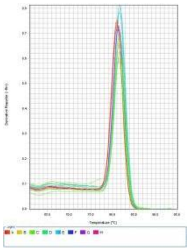

Stand Curve of NADH

Slope: -3.2766 Intercept: 37.502 y=--3.2766x + 37.502 NADH PCR Effidency: 102%

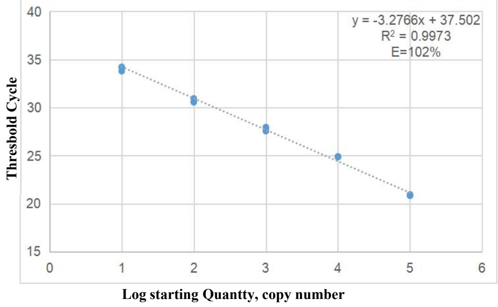

Amplification Plot of NADH

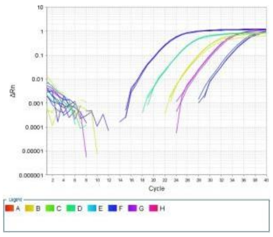

Melt Curve of NADH

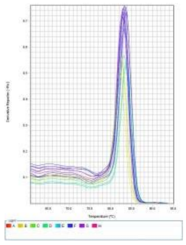

Stand Curve of TUBA

Slope: -3.2374 Intercept: 39.248 y=--3.2374x + 39.248 TUBA PCR Effidency: 103%

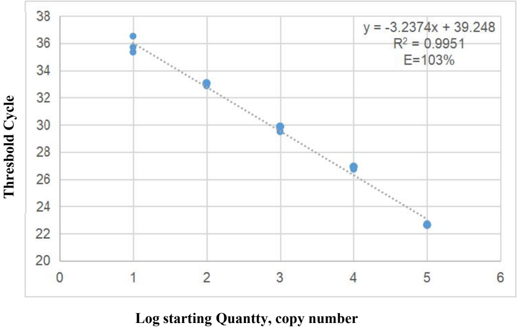

Amplification Plot of TUBA

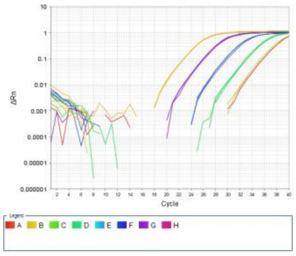

Melt Curve of TUBA

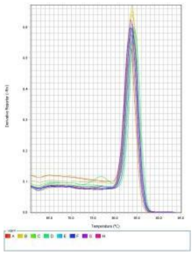

Stand Curve of NDUFA13

Slope: -3.1568 Intercept: 38.159 y=--3.1568x + 38.159 NDUFA13 PCR Effidency: 107%

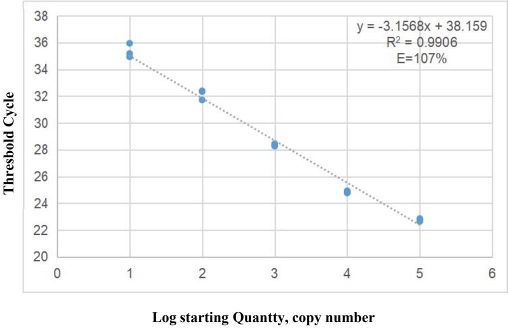

Amplification Plot of NDUFA13

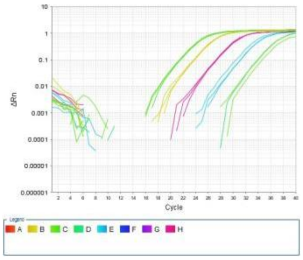

Melt Curve of NDUFA13

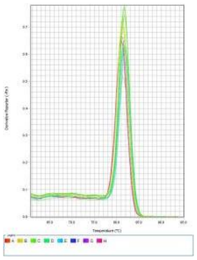

Stand Curve of RPL18A

Slope: -3.3029 Intercept: 33.944 y=--3.3029x + 33.944 RPL18A PCR Effidency: 100%

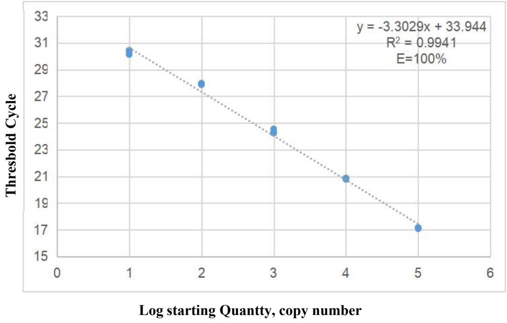

Amplification Plot of RPL18A

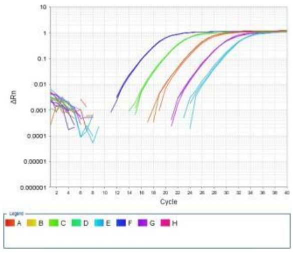

Melt Curve of RPL18A

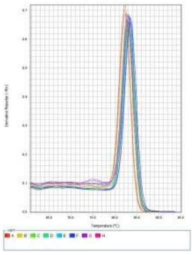

Stand Curve of TUBB

Slope: -3.3152 Intercept: 33.859 y=--3.3152x + 33.859 TUBB PCR Effidency: 100%

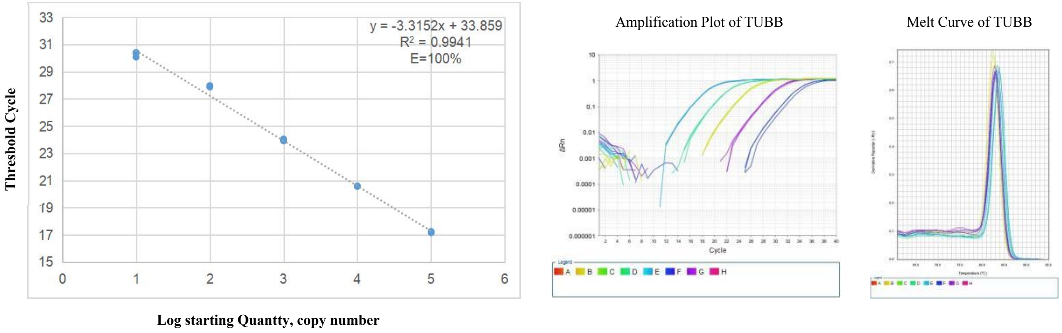

Stand Curve of EF1α

Slope: -3.3416 Intercept: 38.156 y=--3.3416x + 38.156 EF1α PCR Effidency: 99%

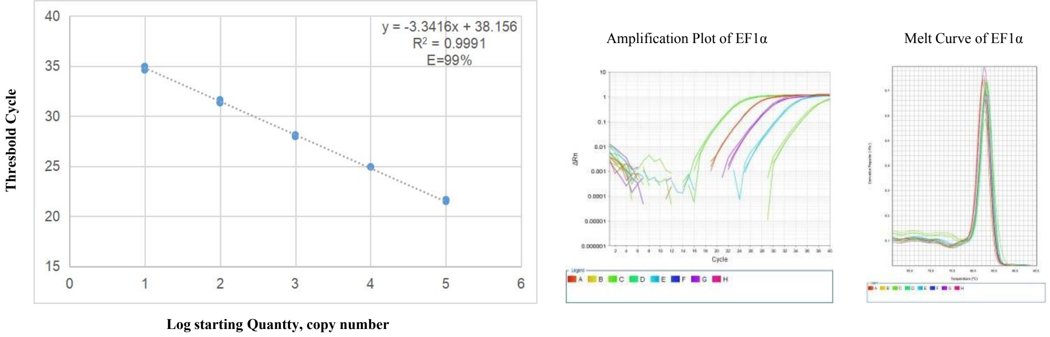

Stand Curve of RPS18

Slope: -3.4252 Intercept: 38.366 y=--3.4252x + 38.366 RPS18 PCR Effidency: 96%

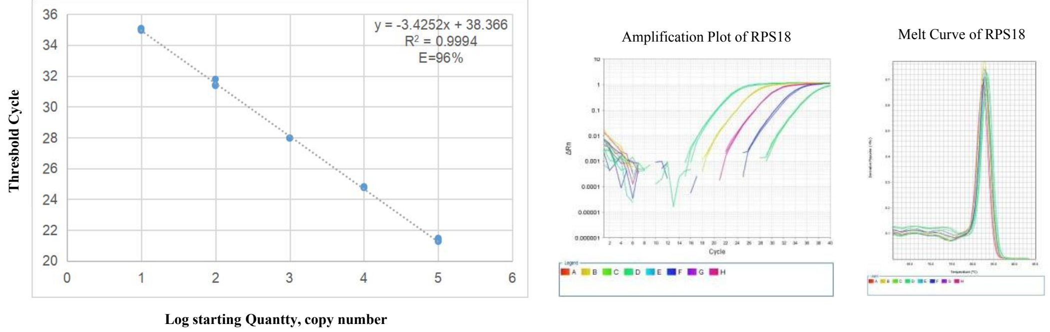

3. Analysis of gene transcription stability

We tested ten candidate housekeeping genes for expression stability in sea cucumber coelomocytes post *V. splendidus*-challenge at five different time points (0, 12, 24, 48, and 72 h) and in five healthy sea cucumber tissues, including coelomocytes, intestines, muscles, tentacles, and respiratory trees with three biological replicates. The raw data of qRT-PCR was performed using boxplot analysis (**Fig. 2**). The results showed Ajβ-actin was the most abundant housekeeping gene

with the lowest mean CT value of 17.66 and 20.90 in both *V. splendidus*-challenged coelomocytes and healthy sea cucumber tissues, respectively. NDUFA13 was the least expressed housekeeping gene with the highest mean CT value of 26.74 and 27.98 in both *V. splendidus*-challenged samples and healthy sea cucumber tissues, respectively.

In this study, we used three algorithms to select better-suited housekeeping genes for higher-accuracy stability rankings. According to geNorm, stability values (M) below 0.5 are considered stable. For *V. splendidus*-challenged sea cucumber coelomocytes, geNorm determined that Ajβ-actin and EF1α were the most stable reference genes, followed by NADH, NDUFA13, TUBB, RPS9, RPS18, and RPL18A. Except for TUBA and GAPD, the stability value (M) of other candidate housekeeping genes is lower than 0.5 (**Fig. 3a**). In tissue samples, geNorm determined that TUBA and NDUFA13 were the most stable reference genes. However, the stability values (M) of other candidate housekeeping genes are higher than 0.5 (**Fig. 3b**). According to NormFinder algorithm analysis, the housekeeping gene with the lowest stability values (M) is the most stable reference gene. NormFinder identified that Ajβ-actin was the most stable reference gene in *V. splendidus*-challenged sea cucumber coelomocytes (**Fig. 4a**), while RPS18 is the most stably expressed in healthy sea cucumber tissues, followed by Ajβ-actin (**Fig. 4b**). BestKeeper calculates standard deviation (SD) to identify stable reference genes with SD values inversely proportional to expression stability. As shown in **Table 2**, the expression of Ajβ-actin is the most stable gene under *V. splendidus* infection, while RPS18 is the most stable reference

gene in healthy sea cucumber tissues. According to the integration of the four algorithm results by RefFinder, Ajβ-actin, EF1α, NDUFA13, and TUBB are the most stable four reference genes under *V. splendidus* infection (**Fig. 5a**), while RPS18, Ajβ-actin, TUBA, and NDUFA13 are the most stable four reference genes in five different tissues (**Fig. 5b**).

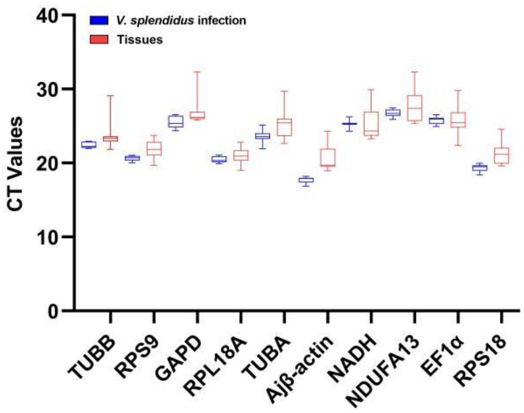

Fig. 2 Boxplot of absolute cycle threshold values for ten candidate reference genes. The median is indicated by a line in each box. Whiskers indicate when the values go down to the smallest value and up to the largest.

a b

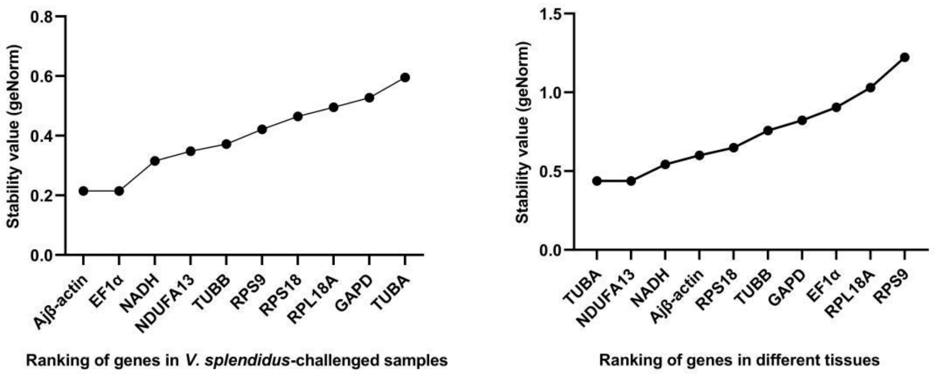

Fig. 3 Expression stability of the candidate reference genes in *V. splendidus*-challenged samples (a) and in different tissues (b) by geNorm.

a

b

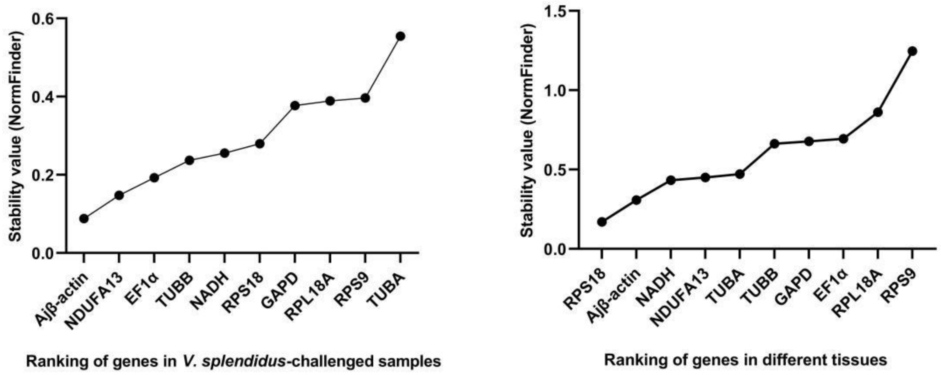

Fig. 4 Expression stability of the candidate reference genes in *V. splendidus*-challenged samples (a) and in different tissues (b) by NormFinder.

Table 2 Expression stability values of the candidate reference genes calculated by BestKeeper

Gene	*V. splendidus* infection		Tissues	
	r	SD	r	SD
TUBB	0.655	±0.303	0.959	±0.760
RPS9	0.014	±0.339	0.538	±1.086
GAPD	0.823	±0.465	0.944	±0.859
RPL18A	0.297	±0.423	0.861	±0.657
TUBA	0.870	±0.537	0.976	±0.543
**Ajβ-actin**	**0.959**	**±0.133**	0.977	±0.449
NADH	0.808	±0.362	0.988	±0.396
NDUFA13	0.918	±0.217	0.990	±0.369
EF1α	0.826	±0.279	0.936	±0.899
RPS18	0.744	±0.375	**0.992**	**±0.238**

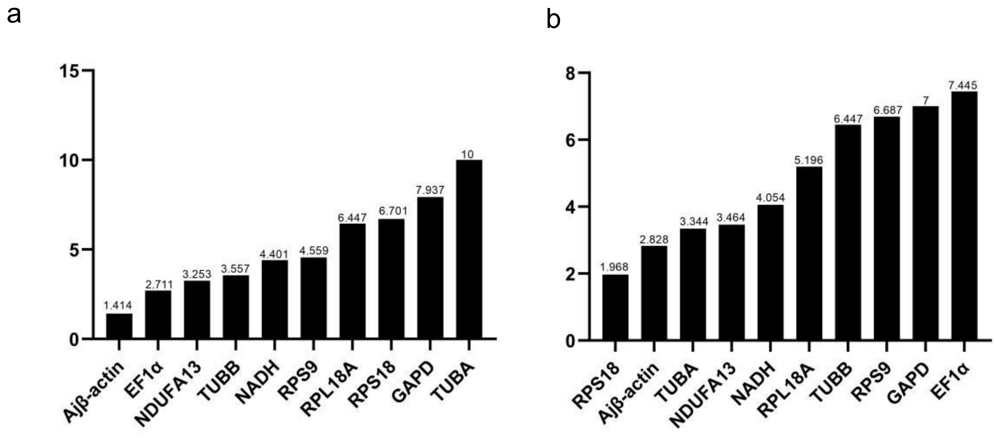

Fig. 5 Expression stability of the candidate reference genes in *V. splendidus*-challenged samples (a) and in different tissues (b) by RefFinder.
