# Supplementary material for: circRNA432 enhances the coelomocyte phagocytosis via regulating the miR-2008-ELMO1 axis in Vibrio splendidus-challenged Apostichopus japonicus
Source: Commun Biol. 2023 Jan 28;6:115. doi: 10.1038/s42003-023-04516-8 (PMC9884281; doi:10.1038/s42003-023-04516-8)
Supplement: Supplementary file 5 — Reporting Summary [file 42003_2023_4516_MOESM5_ESM.pdf]

nature portfolio

Corresponding author(s): COMMSBIO-22-2620B

Last updated by author(s): Jan 12, 2023

Reporting Summary

Nature Portfolio wishes to improve the reproducibility of the work that we publish. This form provides structure for consistency and transparency in reporting. For further information on Nature Portfolio policies, see our Editorial Policies and the Editorial Policy Checklist.

Statistics

For all statistical analyses, confirm that the following items are present in the figure legend, table legend, main text, or Methods section.

n/a	Confirmed	
☐	☒	The exact sample size (*n*) for each experimental group/condition, given as a discrete number and unit of measurement
☐	☒	A statement on whether measurements were taken from distinct samples or whether the same sample was measured repeatedly
☐	☒	The statistical test(s) used AND whether they are one- or two-sided<br>*Only common tests should be described solely by name; describe more complex techniques in the Methods section.*
☒	☐	A description of all covariates tested
☐	☒	A description of any assumptions or corrections, such as tests of normality and adjustment for multiple comparisons
☐	☒	A full description of the statistical parameters including central tendency (e.g. means) or other basic estimates (e.g. regression coefficient) AND variation (e.g. standard deviation) or associated estimates of uncertainty (e.g. confidence intervals)
☐	☒	For null hypothesis testing, the test statistic (e.g. *F*, *t*, *r*) with confidence intervals, effect sizes, degrees of freedom and *P* value noted<br>*Give P values as exact values whenever suitable.*
☒	☐	For Bayesian analysis, information on the choice of priors and Markov chain Monte Carlo settings
☐	☒	For hierarchical and complex designs, identification of the appropriate level for tests and full reporting of outcomes
☐	☒	Estimates of effect sizes (e.g. Cohen's *d*, Pearson's *r*), indicating how they were calculated

Our web collection on statistics for biologists contains articles on many of the points above.

Software and code

Policy information about availability of computer code

Data collection	No computer code was involved in this study.
Data analysis	No commercial, open source and custom code were involved in this study.

For manuscripts utilizing custom algorithms or software that are central to the research but not yet described in published literature, software must be made available to editors and reviewers. We strongly encourage code deposition in a community repository (e.g. GitHub). See the Nature Portfolio guidelines for submitting code & software for further information.

Data

Policy information about availability of data

All manuscripts must include a data availability statement. This statement should provide the following information, where applicable:

- Accession codes, unique identifiers, or web links for publicly available datasets
- A description of any restrictions on data availability
- For clinical datasets or third party data, please ensure that the statement adheres to our policy

Provide your data availability statement here.

Human research participants

Policy information about studies involving human research participants and Sex and Gender in Research.

Reporting on sex and gender	No human research was involved in this study.
Population characteristics	No human research was involved in this study.
Recruitment	No human research was involved in this study.
Ethics oversight	No human research was involved in this study.

Note that full information on the approval of the study protocol must also be provided in the manuscript.

Field-specific reporting

Please select the one below that is the best fit for your research. If you are not sure, read the appropriate sections before making your selection.

☒ Life sciences ☐ Behavioural & social sciences ☐ Ecological, evolutionary & environmental sciences

For a reference copy of the document with all sections, see nature.com/documents/nr-reporting-summary-flat.pdf

Life sciences study design

All studies must disclose on these points even when the disclosure is negative.

Sample size	Healthy adult sea cucumber A. japonicus (weight: 120 ± 5 g).
Data exclusions	No data were exclude from the analyses in this study.
Replication	Each experiment was performed for three times, and all attempts at replication were successfullly.
Randomization	The experimental animals were randomly collected from each group in this study.
Blinding	The investigators were blinded to group allocation during data collection or analysis in this study.

Reporting for specific materials, systems and methods

We require information from authors about some types of materials, experimental systems and methods used in many studies. Here, indicate whether each material, system or method listed is relevant to your study. If you are not sure if a list item applies to your research, read the appropriate section before selecting a response.

Materials & experimental systems

n/a	Involved in the study	
☐	☒	Antibodies
☐	☒	Eukaryotic cell lines
☒	☐	Palaeontology and archaeology
☐	☒	Animals and other organisms
☒	☐	Clinical data
☒	☐	Dual use research of concern

Methods

n/a	Involved in the study	
☒	☐	ChIP-seq
☐	☒	Flow cytometry
☒	☐	MRI-based neuroimaging

Antibodies

Antibodies used	Mouse against A. japonicus ELMO1 polyclonal antibody was performed by ourselves. The commercial antibodies: HRP-conjugated Goat Anti-Mouse IgG (Beyotime D110087-0100, Shanghai, China) , HRP-conjugated Goat Anti-Rabbit IgG (Beyotime D110058-0100, Shanghai, China), β-actin polyclonal antibody (Abmart M20027S, Shanghai, China), Argonaute 2 (AGO2) primary monoclonal antibody (Abmart ab186733, Shanghai, China), and anti-GFP antibody (Abcam ab6556, Shanghai, China)
Validation	For the validation of ELMO1 antibody specificity, we performed the recombinant and purified Elmo1 protein, total protein of sea cucumber coelomocytes, and other unrelated proteins. For the validation of β-actin antibody specificity, this antibody was shown to

have the reactivity with the β-actin of Human, Rabbit, and Mouse, as described in the website (http://www.kleanab.com/English/productDetail.jsp?id=5377).

Eukaryotic cell lines

Policy information about cell lines and Sex and Gender in Research

Cell line source(s)	Hela cell line
Authentication	The Hela cell line was purchased from Zhejiang Meisen Cell Technology Co., Ltd.(https://www.ctcc.online/ProductCenter/info.aspx?itemid=5526)
Mycoplasma contamination	We confirmed that all cell lines tested negative for mycoplasma contamination.
Commonly misidentified lines (See ICLAC register)	We did not name any commonly misidentified cell lines used in the study.

Animals and other research organisms

Policy information about studies involving animals; ARRIVE guidelines recommended for reporting animal research, and Sex and Gender in Research

Laboratory animals	Sea cucumber, A. japonicus, two years old; mouse, Balb/C, 4-5 weeks old.
Wild animals	No wild animals were involved in this study.
Reporting on sex	The sex of the experimental animals was not considered in this study.
Field-collected samples	The study did not involve samples collected from the field.
Ethics oversight	The sea cucumbers A. japonicus used in this work were commercially cultured animals, and all experiments were conducted in accordance with the recommendations in the Guide for the Care and Use of Laboratory Animals of the National Institutes of Health. The study protocol was approved by the Experimental Animal Ethics Committee of Ningbo University, China (No. NBU-ES-2021-11180).

Note that full information on the approval of the study protocol must also be provided in the manuscript.

Flow Cytometry

Plots

Confirm that:

☒ The axis labels state the marker and fluorochrome used (e.g. CD4-FITC).

☒ The axis scales are clearly visible. Include numbers along axes only for bottom left plot of group (a 'group' is an analysis of identical markers).

☒ All plots are contour plots with outliers or pseudocolor plots.

☒ A numerical value for number of cells or percentage (with statistics) is provided.

Methodology

Sample preparation	The coelomic fluids were collected from the individuals in each tank and centrifuged at 800 g, 4°C for 5 min to harvest coelomocytes. For cell phagocytosis assay, the sea cucumbers were injected with FITC-labeled V. splendidus, and the coelomocytes were harvested after V. splendidus challenge, and the phagocytic activity was examined by a Flow Cytometer. For spatial expression analysis, five tissues including coelomocytes, muscle, tentacle, respiratory trees, and intestine were collected from the untreated sea cucumbers. These tissues were homogenized into powder in liquid nitrogen using a mortar and a pestle.
Instrument	The phagocytic activity was detected by the MACSQuant Analyzer 10 (Miltenyi Biotec, Bergisch Gladbach, Germany) in this study.
Software	Flowjo V10 was used to analysis the flow cytometry data in this study.
Cell population abundance	After 48 h of in vivo interference, 200 μL of FITC-labeled V. s (OD600 = 1.0) was injected into sea cucumbers. Coelomocytes were harvested after V. s challenge for 2 h. After cells were washed 5 times with PBS, the cells were diluted to 10,000 cells with 200 μL PBS, filtered with 300-mesh gauze, and placed in a flowmeter tube. Flow cytometry (Thermo Scientific, Madison, WI, USA) was used to detect cell phagocytosis. All viable cells were gated based on FSC and SSC plot, which account for 85~95% of all cells, and dead cells/deris were excluded from the analysis. Gated cells were furhter analyzed on FITC fluorescent intensity and as shown in Fig 7C, cells phagocytosing targets exhibited increased fluorescent intensity and values > 10^1 on the x axis were considered as positive cells in our analysis.

Gating strategy

All viable cells were gated based on FSC and SSC plot, which account for 85~95% of all cells, and dead cells/deris were excluded from the analysis. Gated cells were furhter analyzed on FITC fluorescent intensity and as shown in Fig 7C, cells phagocytosing targets exhibited increased fluorescent intensity and values > 10^1 on the x axis were considered as positive cells in our analysis.

☐ Tick this box to confirm that a figure exemplifying the gating strategy is provided in the Supplementary Information.
